# Environmental change or choice during early rearing improves behavioural adaptability in laying hen chicks

**DOI:** 10.1038/s41598-023-33212-0

**Published:** 2023-04-15

**Authors:** Lena Skånberg, Ruth C. Newberry, Inma Estevez, Linda J. Keeling

**Affiliations:** 1 Department of Animal Environment and Health, Swedish University of Agricultural Sciences, P.O. Box 7068, 750 07 Uppsala, Sweden; 2 Department of Animal and Aquacultural Sciences, Faculty of Biosciences, Norwegian University of Life Sciences, 1432 Ås, Norway; 3 Department of Animal Production, NEIKER Basque Research and Technology Alliance (BRTA) Basque Institute for Agricultural Research, 01080 Vitoria-Gasteiz, Spain; 4 IKERBASQUE Basque Foundation for Science, Euskadi Plaza 5, 48009 Bilbao, Spain

**Keywords:** Animal behaviour, Zoology

## Abstract

Laying hens are typically moved to a novel environment after rearing, requiring adaptability to cope with change. We hypothesized that the standard rearing of laying hen chicks, in non-changing environments with limited choices (a single variant of each resource), impairs their ability to learn new routines, use new equipment and exploit new resources. On the contrary, rearing in a changing environment that also offers a choice of resource variants could better prepare chicks for the unexpected. To explore this hypothesis, environmental change and choice were manipulated in a 2 × 2 factorial experiment. Compared to standard rearing, greater change during early rearing, through repeatedly swapping litter and perch types, reduced initial freezing when exposed to a novel environment suggesting a lower fear response. Greater choice during rearing, through simultaneous access to multiple litter and perch types, resulted in shorter latencies to solve a detour task, more movement in novel environments and less spatial clustering, suggesting improved spatial skills and higher exploration. However, combining both change and choice did not generally result in greater improvement relative to providing one or the other alone. We conclude that environmental change and choice during rearing have different positive but non-synergistic effects on later adaptability potential.

## Introduction

At certain points in their life, domestic animals and captive wildlife are often transferred between different environments. Such moves involve exposure to novel resources, routines and situations, which can be stressful and imply a welfare risk. For example, when young laying hens (*Gallus gallus domesticus*) are transferred from a relatively simple rearing environment to a more complex, multi-tiered adult housing system, they can suffer injuries and high mortality^1,2^. A recommendation for farm animals is to match the type of housing system used in the rearing and adult periods as a way to reduce potential negative outcomes from such transitions^3,4^. However, even if the housing system is similar, the transition from one facility to another still involves some environmental change. The difficulties encountered by animals following the transition may persist throughout adult life, affecting both welfare and reproductive performance^4–6^. Improved ability to cope with change would enable a smoother transition from the rearing facilities to the subsequent environments. Beside the practical relevance of reducing stress when transferred to a new environment, there is a need for improved understanding of general principles for promoting the adaptability potential of captive animals during rearing^7,8^.

Experience gained during the first weeks of life, a sensitive period for brain development, can have long lasting and often irreversible effects on behaviour and cognitive abilities^9^. Plasticity during development allows the forming of abilities that can contribute to improved health and fitness when adult in a future environment^10^. Captive animals are often reared in static environments that offer limited behavioural opportunities. Compared to the complex, dynamic natural environments of their wild ancestors, young laying hens are typically exposed to a low degree of stimulation from their rearing environment, which could limit their ability to learn new routines, use new equipment, and exploit new resources encountered later in life. In contrast, experience of mildly stressful situations or spatially complex environments during early life may stimulate the development of adaptations that will be advantageous, facilitating the adjustments to novel environments. For example, in rodents, mild stress experienced early in life has been shown to lead to improved stress coping later in life^11,12^. Similarly, in chicks, early cold stress led to a shorter latency to initiate movement in a novel environment at five weeks of age, suggesting that stressed chicks were less frightened by the novelty^13^. However, early cold stress exposure also came with negative effects, including slower first use of elevated structures and suppressed immune responses^14^. Interestingly, negative cold stress effects were ameliorated in birds exposed to spatially complex rearing environments with access to additional perches and shelter^14^, suggesting an interaction between the effects of early stress and environmental complexity. With regards to environmental complexity, rearing with (versus without) access to perches during the first four weeks of life improves laying hens’ later ability to move in three dimensional space^15^. For example, aviary-reared hens are faster locating a reward in a spatial task and have greater working memory compared to individuals reared in a simple cage environment^16^. These results may be reflecting increased neural activation in the hippocampus, a brain region highly associated with spatial skills and navigation, which is seen already in day-old chicks exposed to spatially complex environments^17,18^. Thus it can be suggested that early exposure to mild stress and spatial complexity may contribute to an improved ability to cope with future stressful situations and greater ability to make the most of opportunities when they arise.

Two more aspects, predictability and controllability of resources available in the environment can play important roles in coping with stress^19–21^. It is possible that the positive effects of early stress and spatial complexity described earlier are a consequence of how young animals’ experience the predictability and controllability of their environment. This experience influences stress states and, in turn, behavioural^22^ and physiological adaptability^23,24^. Meagher^25^ suggests that some level of unpredictability in the environment could be beneficial for reducing boredom in captive animals and that any stress arising from this unpredictability could be reduced by adding choice to the environment. Effects of unpredictability early in life have been investigated following increasing levels of change in the home environment, by moving^26^ and exchanging objects^27^, or changing routines^28^. Choice, on the other hand, is tightly connected to the perception of controllability^29^. Increased choice can be given by setting up environmental gradients, thereby increasing spatial complexity^30^. Given these connections, it is possible that greater levels of environmental change and choice when young could be linked to improvements in coping ability.

**Figure 1:**
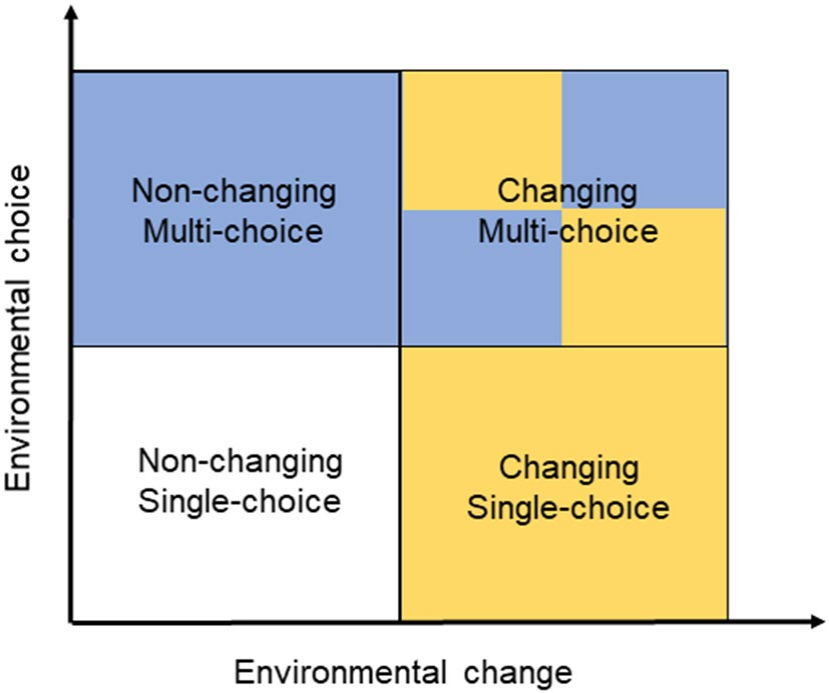
Four treatments varying in levels of environmental change (Non-changing/Changing) and choice (Single/Multi). They represent two different types of stimulation (Change or choice) intended to manipulate experienced predictability and controllability, respectively. The 2 × 2 factorial design allowed us to explore the effects of change and choice during rearing on the welfare of laying hen chicks and their behavioural adaptability in novel situations. The combination of a Non-changing and Single-choice environment (bottom left quadrant) was considered to represent the standard rearing condition.

Litter and perches are valuable resources for laying hen chicks^4^ and they differentiate between different types already from the first days of life^31^. Standard rearing environments for laying hen chicks usually offer only a single type of litter and a single type of perch throughout the rearing period. This results in a simple and non-changing environment with limited possibilities for individual choice (i.e., a predictable environment with low controllability), which is not optimal according to Meagher^25^. Environmental change and choice could be altered by manipulating the litter and perch types available to laying hen chicks across time and space. The level of environmental change can be increased compared to the standard rearing environment by repeatedly changing the type of litter and perch offered over time, producing a more unpredictable environment. The level of environmental choice could be increased compared to the standard rearing environment by offering multiple types of litter and perches in different locations of the pen, thereby increasing spatial complexity, and giving chicks more control over the types of substrates used for performing different activities. Both change and choice could be increased by simultaneously offering multiple litter and perch types, as well as changing their location over time. In this study, we explored effects of early exposure of domestic chicks to different levels of environmental change and choice in a 2 × 2 factorial experiment (Fig. 1) during their first four weeks of rearing.

Our first goal was to assess how different levels of change and choice would affect chick welfare during imposition of the rearing treatments. This was necessary to evaluate the extent to which chicks were affected by the different treatments, as indicated using behavioural measures of stress (for physiological measures, see^32^), comfort^33^ and agency^30,34^. Since agency reflects motivation for environmental engagement, it can be associated with behaviours connected to environmental interaction, such as foraging and movement. Physiological or psychological stressors activate the hypothalamo-pituitary-adrenal (HPA) axis, which plays an important role in regulating behavioural expression in bird^35^. The HPA-axis is already fully functional on day 1 post-hatch in laying hen chicks^36^. Thus, the behaviour of young chicks can reflect central stress mediation from an early phase of development. Our second goal was to evaluate the impact of the treatments on behavioural adaptability, which is relevant to understanding how the rearing treatments could affect chick ability to cope with a transition to a novel environment. To achieve this second goal, we conducted two standardised novelty challenge tests. The Novel pen test evaluated group-level acute behavioural responses following transition to a novel environment with unfamiliar resource types. The Multivariate behavioural test was used to investigate fearfulness, spatial abilities and exploration simultaneously at the individual level^13^.

We hypothesised that, when compared to a treatment representing standard rearing conditions; (1) a higher level of environmental change, achieved by repeated changes of litter and perch types, would result in mild stress during rearing but provide coping experience, thereby reducing fearfulness and leading to greater adaptability when challenged with novelty^11,12^; (2) a higher level of environmental choice, achieved by simultaneously providing a variety of litter and perch types, would help to fulfil motivation for behavioural activities such as foraging and dustbathing leading to greater comfort and agency, along with enhancement of spatial skills^17,37^ including practicing the different bodily actions needed to land and balance on the different perch types^15,31^; while (3) higher levels of both environmental change and choice would enhance both adaptability and agency, and lead to the most advantageous outcomes in terms of both welfare during rearing and adaptability in response to novelty.

Predictions arising from these hypotheses were that, compared to chicks in the standard rearing treatment; (1) chicks exposed to higher levels of environmental change would exhibit higher levels of behaviours indicative of mild stress (e.g. elevated vigilance, pecking at conspecifics and spatial clustering directly after each change), but also more rapid exploration and location of novel resources in the novelty challenge tests; and (2) chicks exposed to higher levels of environmental choice would exhibit higher levels of behaviours indicative of comfort (e.g. preening) and active engagement with their environment indicating higher agency (e.g. foraging, dustbathing, movement, play and reduced behavioural synchronization); while (3) chicks exposed to higher levels of both environmental change and choice would show signs of mild stress, comfort, and active environmental engagement during rearing, as well as being quicker to move and exploit new resources in the novelty challenges.

## Results

**Figure 2:**
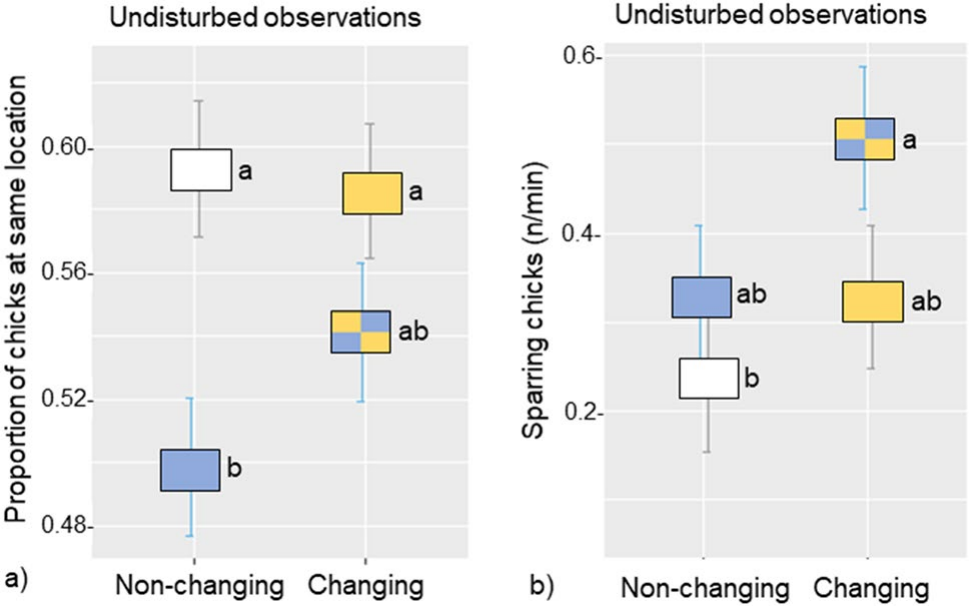
Treatment differences in the rearing pens under undisturbed conditions were found for (**a**) spatial clustering, calculated as the estimated marginal mean (emmean) ± SE proportion of chicks at the same resource location per instantaneous scan and (**b**) emmean ± SE number of chicks sparring per minute per pen. The treatment combinations were the standard rearing condition Non-changing*Single-choice (white), Non-changing*Multi-choice (blue), Changing*Single-choice (yellow), and Changing*Multi-choice (blue and yellow). Treatments with no letter in common, a or b, were significantly different (*P* ≤ 0.05).

**Figure 3:**
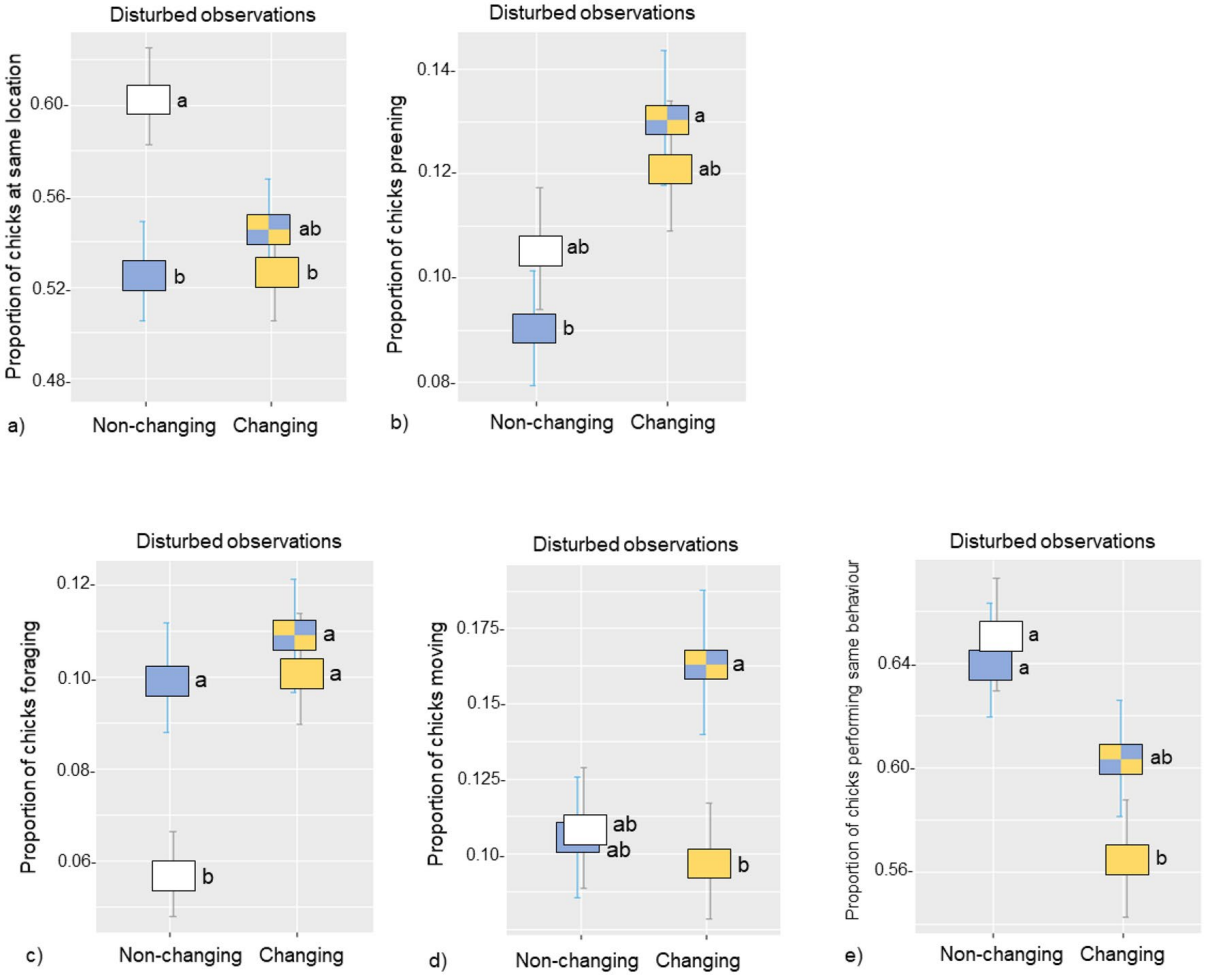
Treatment differences in the rearing pens following disturbance were found in the emmean ± SE proportion of chicks/scan that showed (**a**) spatial clustering (calculated as the proportion of chicks at the same location per instantaneous scan), (**b**) preening, (**c**) foraging, (**d**) moving and (**e**) behavioural synchronization (calculated as the proportion of chicks performing the same behaviour per instantaneous scan). The treatment combinations were the standard rearing condition Non-changing*Single-choice (white), Non-changing*Multi-choice (blue), Changing*Single-choice (yellow), and Changing*Multi-choice (blue and yellow). Disturbed observations were carried out in the first 4 h after changes were made in Changing pens (and the equivalent period in Non-changing pens). Treatments with no letter in common, a or b, were significantly different (*P* ≤ 0.05).

**Figure 4:**
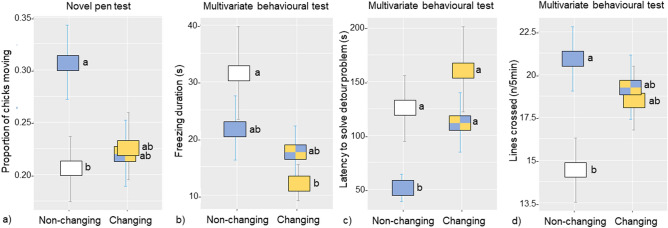
Treatment differences found in the novelty challenge tests. In the Novel pen, a treatment effect was found for (**a**) proportion of chicks moving per instantaneous scan. In the Multivariate behavioural test, treatment effects were found for all behaviour measures, including (**b**) duration of freezing behaviour, (**c**) time to exit the start box by solving the detour task, and (**d**) number of lines crossed during a 5-min period in the open arena (see Fig. 6b). The treatment combinations were the standard rearing condition Non-changing*Single-choice (white), Non-changing*Multi-choice (blue), Changing*Single-choice (yellow), and Changing*Multi-choice (blue and yellow). Values presented are emmeans ± SE. Treatments with no letter in common, a or b, were significantly different (*P* ≤ 0.05).

Results are presented in graphs illustrating the interaction between the two levels of environmental change (Non-changing/Changing) and the two levels of environmental choice (Single/Multi-choice). First, we present behaviour observed in the rearing pens where differences were found between treatments during observation periods both under Undisturbed conditions (Fig. 2) and within the first four hours after the disturbance of a person entering the pen to change (or not) the litter and perches (Disturbed conditions; Fig. 3). Secondly, we present results from novelty challenges (Fig. 4) involving transition of groups to a Novel pen and individuals to a Multivariate behavioural test. A summary of significant comparisons is presented in Table 1, while behavioural variables without significant treatment differences are presented in Supplementary Information (Table 1).

**Table 1:** Summary of the significant treatment differences, taking the standard rearing condition, Non-changing*Single-choice (white) as the reference versus Changing*Single-choice (yellow), Non-changing*Multi-choice (blue) and Changing*Multi-choice (yellow and blue).

Treatment comparisons	Resulted in
Hhigher level of Change vs Standard	Less spatial clustering (Disturbed): z = − 2.522, *P* = 0.012 (Fig. 3a) Less synchronization (Disturbed): z = − 2.733, *P* = 0.006 (Fig. 3e) More foraging (Disturbed): z = 2.90, *P* = 0.004 (Fig. 3c) Reduced freezing duration (Multivariate behavioural test): t = − 2.58, *P* = 0.024 (Fig. 4b)
Higher level of Choice vs Standard	Less spatial clustering (Undisturbed): z = − 3.066, *P* = 0.002 (Fig. 2a) Less spatial clustering (Disturbed): z = − 2.522, *P* = 0.012 (Fig. 3a) More foraging (Disturbed): z = 2.794, *P* = 0.005 (Fig. 3c) More movement (Novel pen test): z = 2.162, *P* = 0.031 (Fig. 4a) Shorter latency to solve detour task (Multivariate behavioural test): t = − 2.56, *P* = 0.025 (Fig. 4c) More lines crossed (Multivariate behavioural test): t = 2.45, *P* = 0.031 (Fig. 4d)
Higher levels of Change and Choice vs Standard	More sparring (Undisturbed): t = 2.41, *P* = 0.024 (Fig. 2b) More foraging (Disturbed): z = 3.283, *P* = 0.001 (Fig. 3c)
Higher level of Change in a Multi-Choice environment	More preening (Disturbed): z = 2.370, *P* = 0.018 (Fig. 3b) Longer latency to solve detour task (Multivariate behavioural test): t = 2.25, *P* = 0.044 (Fig. 4c)
Higher level of Choice in a Changing environment	More moving (Disturbed): z = 2.117, *P* = 0.034 (Fig. 3e)
Higher level of Choice vs higher level of Change	Less spatial clustering (Undisturbed): z = − 2.832, *P* = 0.005 (Fig. 2a) More synchronization (Disturbed): z = 2.418, *P* = 0.016 (Fig. 3e) Shorter latency to solve detour task (Multivariate behaviour test): t = − 3.297, *P* = 0.006 (Fig. 4c)

It is specified in parenthesis whether behavioural differences occurred in the rearing pens during Undisturbed conditions or during Disturbed conditions (the first 4 h after changes were made in the Changing pens or the mere human entry in the Non-changing pens), during the group observations in a Novel pen, or during individual testing in the Multivariate behavioural test and also to which figure graph the results refer.

### Treatment differences in rearing pens when undisturbed

Treatment differences in spatial clustering (indicating mild stress, Fig. 2a) and sparring behaviour (social play, Fig. 2b) were found in observations on undisturbed chicks during rearing. See Table 1 for a summary of these differences.

### Treatment differences in rearing pens following disturbance

The majority of the treatment differences during rearing were detected from observations made in the first four hours following disturbance associated with making environmental changes in the Changing pens (and the equivalent period in Non-changing pens). Differences were found in spatial clustering (indicating mild stress, Fig. 3a) as well as the average proportion of chicks preening (comfort, Fig. 3b), foraging, moving and behavioural synchronization (indicating environmental engagement, Fig. 3c,d,e). See Table 1 for a summary of these differences.

### Novelty challenge tests

In the Novel pen test, in which groups of 10 chicks were placed in a novel pen and observed for one hour, treatment differences were found in the proportion of chicks moving per scan (Fig. 4a). No differences were found in the other measured variable, which was the average proportion of chicks exploiting novel resources (substrates, feed platform, perches) in the pen (Table 1). In the Multivariate behavioural test, in which chicks were tested individually, treatment differences were found for all three phases of the test. Behavioural differences were found in freezing duration (latency to move any body part after being placed in the start box; Fig. 4b), time to exit the start box and solve the detour task after the first freezing bout (Fig. 4c), and number of lines crossed during 5 min in the open arena (Fig. 4d). See Table 1 for a summary of these differences.

## Discussion

Our study design revealed the effects of varying levels of environmental change and choice during early rearing on laying hen chicks’ welfare and adaptability. During undisturbed observations, chicks with greater environmental choice showed less spatial clustering, while chicks having greater environmental change and choice showed more social play behaviour (sparring). Following disturbance, associated with changing the litter and perches in changing pens, or the mere entry of a person into non-changing pens, chicks having greater environmental change, choice, or change and choice showed more foraging behaviour. The responses of the chicks in the two novelty challenge tests, the Novel pen (group) and the Multivariate behavioural test (individual), were intended to indicate how chicks from the various rearing environments might adapt to a change of environment later in life. Chicks reared with a higher level of change had a shorter initial freezing duration when placed in the Multivariate behavioural test. Chicks reared with more choice moved more in both novelty challenge tests, and were quicker to solve the detour task in the Multivariate behavioural test. This improved adaptability was not shown by chicks reared with higher levels of both change and choice, making their responses to the novelty challenges no different to those of birds reared in the standard pens.

### Higher level of environmental change

Behavioural differences between chicks that had only one litter and perch type, but that varied in the level of environmental change (Non-changing*Single-choice versus Changing*Single-choice) were found during the disturbed period and in the Multivariate behavioural test. Following the first hours after the exchange of the litter and perch type, chicks showed less spatial clustering, less behavioural synchronization and more foraging, compared to chicks only having the human visit. This implies a higher level of environmental engagement, with chicks exploring the new litter and perch type and being more dispersed as a result of the increased activity^38^. We had predicted that birds may experience the changing environment as somewhat stressful and that this would be reflected in increased vigilance, pecking at conspecifics and spatial clustering directly following changes. That this was not the case, and opposite effects to those predicted were observed for spatial clustering, suggests that the imposed level of change was perceived as stimulating rather than aversive. Further, the lack of differences in behaviour at times other than the first four hours after each change (i.e. during undisturbed observations) shows that the chicks rapidly adapted to the changes.

In the Multivariate behavioural test, the lower freezing duration of chicks from the Changing*Single-choice treatment compared to the standard-reared chicks (Non-changing*Single-choice treatment) implies that they were less fearful^39^. This result supports the idea that the experience of novelty that is not excessive or uncontrollable leads to reduced fearfulness^39^, again suggesting that any stress arising from the changing environment was mild. For chicks living in a less complex environment, our results support the idea that regular changes in the environment (e.g. by swapping the type of litter and perch available) have welfare benefits. The increased environmental engagement, even if only short term, can be associated with a lower risk of boredom, as predicted by Meagher^25^. Reduced fearfulness of birds reared in a changing environment could lead to a smoother transition between rearing and adult layer facilities and may also lead to a lower prevalence of severe feather pecking^40^, a major welfare problem in today’s laying hen environments. It could also lead to reduced muscle and skeletal damage related to collision during sudden escape attempts^41^. It is possible that, according to our hypothesis, the changes we imposed resulted in mild stress that improved stress resilience^11,12^, leading to the reduced freezing response. Alternatively, the increased environmental engagement stimulated by the procedure of swapping litter and perch types reflected positive learning experiences about novelty leading to the reduced reaction when placed in the start box of the Multivariate behavioural test.

### Higher level of environmental choice

Behavioural differences between chicks from the non-changing pens varying in the level of environmental choice (Non-changing*Single-choice versus Non-changing*Multi-choice) were found in all parts of this study. Chicks with a choice of four litter and perch types that were not relocated (Non-changing*Multi-choice) showed less spatial clustering in their pens compared to standard-reared chicks (Non-changing*Single-choice) throughout rearing. This, together with the increased foraging during the observations following disturbances, could support greater active engagement with the environment and thereby development of greater agency, as hypothesised. But contrary to our hypothesis, neither play nor dustbathing (indicating active engagement and a positive response to choice) was increased. This could be due to the relative rarity of these behaviours. We also found no support for the prediction of a higher level of the comfort behaviour, estimated by occurrences of preening. The reduced spatial clustering observed in the Multi-choice pens is unlikely to be explained by an increased need to avoid competition, feather pecks or aggressive behaviour in this treatment. All chicks could fit into the same litter tray or onto the same perch and, thus, exploit the same resource type at the same time^42^. Also, we saw very few aggressive pecks, and aggression is known to be low in chicks of this age^43,44^. Furthermore, no treatment differences were detected in severe feather pecking. Instead, the reduced spatial clustering, and that chicks more often crossed into parts of the open area in the Multivariate behavioural test where they had no visual contact with their pen mates, could indicate higher exploration connected to greater risk-taking^45,46^.

The higher degree of movement in both the Novel pen and the Multivariate behavioural test by chicks reared with a higher level of choice did not reflect movement in their rearing pens, which did not differ from that in the standard treatment. Rather, movement can be related to exploration and further reflect increased environmental engagement according to our predictions. Furthermore, that chicks reared with choice had shorter latencies to solve the detour component of the Multivariate behavioural test supports our hypothesis about improved spatial skills, given that the outcome of a detour task reflects spatial abilities^47,48^. The detour task has also been suggested to reflect inhibitory control, as chicks have to inhibit their tendency to directly approach their goal in order to reach it^26,49^. That a spatially more complex rearing environment results in improved spatial skills is supported by previous research^13,15,16,32^, with possible links to neural activity in the hippocampus^17,18^. However, the shorter latency to solve the detour task could also be linked to a generally improved ability to solve problems as a consequence of living in a more enriched environment (reviewed by Zentall^50^). It is also possible that the usage of different variants of resources could have led to a positive feed-back loop whereby positive experiences from choosing different resource types resulted in a more optimistic outlook, as reported in animals living in more enriched or spatially complex environments^13,50^. This could explain the chicks’ higher likelihood of exploring a novel environment as well as their shorter latency to find their way out of the box in the detour task. However, since the detour task was a spatial task, we cannot exclude the possibility that the potentially improved problem-solving ability was limited to improved spatial skills. In the future, it would be interesting to test the ability of chicks reared in a multi-choice environment to solve other types of problems.

Our results indicate that rearing chicks in an environment that provides multiple litter and perch types leads to increased exploration, possibly also involving greater risk-taking, as well as improved spatial skills. Compared to their wild ancestor, the red jungle fowl, domestic fowl show impaired spatial learning^28^ and are less willing to explore for hidden food sources^51^ implying an even greater need to promote these abilities. Exploration in laying hens has been associated with lower mortality, lower feather pecking levels and less fear of humans^52,53^, which would all be advantageous in a poultry farm setting. More risk-taking behaviour in a production setting where predators are absent would facilitate rapid adaptation as they would be more likely to explore and locate the resources. Ability to navigate in a novel multi-dimensional space and to locate feed, nests and water is crucial when birds are transferred to an aviary-style laying house^1,54^*.* That an environment with a higher level of environmental choice results in birds more able to acquire information and enhance skills fits with the predictions of $\check{S}$ pinka^30^. Our results suggest that a more spatially complex rearing environment better prepared chicks for future challenges by promoting behavioural adaptability that would be advantageous in a future spatially complex environment.

### Higher level of environmental change and choice combined

Behavioural differences between chicks reared with higher levels of change and choice (Changing*Multi-choice) compared to standard-reared chicks (Non-changing*Single-choice) were only found in the rearing pen observations. The chicks reared with a combination of increased change and choice (Changing*Multi-choice), like those reared with only increased change (Changing*Single-choice) or only increased choice (Non-changing*Multi-choice), showed increased foraging in association with the disturbance compared to standard-reared chicks. Other than this, the only other behavioural difference from the standard-reared chicks was a higher number of sparring bouts, considered to be a type of play fighting behaviour, in the undisturbed rearing pen observations. Sparring in domestic fowl is most pronounced in chicks during the first weeks of life, before aggressive behaviour emerges^55,56^. Play is generally considered an indicator of good welfare^57^ and, therefore, could imply that the provision of both environmental change and choice had a positive impact on the welfare of the chicks during rearing.

Contrary to our hypothesis, we did not find a generally beneficial synergistic effect of providing both change and choice together during rearing. The results from this treatment (Changing*Multi-choice) were either similar to providing only change (Changing*Single-choice) or only choice (Non-changing*Multi-choice), or intermediate between them, or could even be worse. This latter result occurred in the case of latency to solve the detour task in the Multivariate behavioural test, which was longer for chicks reared with both change and choice than it was for chicks reared only with a higher level of choice. This might be due to a difference in the manner in which environmental change was imposed in combination with choice. In the Changing*Multi-choice treatment, change was accomplished through relocation of the resource variants in space, whereas in the Changing*Single-choice treatment, variants were exchanged for new types over time. Lack of previous experience of repeated exposure to novel resource variants could explain why the freezing duration in the Multivariate behavioural test was not lower among chicks reared with both change and choice compared to the standard reared chicks. That the environmental choices were repeatedly relocated in the Changing*Multi-choice treatment may have also disrupted the positive effects of exposure to a variety of litter and perch types. Laying hens have a characteristic individual pattern of moving between different resources in the pen over the course of each day^58^. Considering that a higher level of choice may increase the experience of controllability^29^, the repeated relocation of the resources may have decreased the sense of control following each relocation. Supporting this, we found more preening following disturbance in the treatment with change and choice than we did in the treatment with choice alone. Preening can either be connected with comfort and relaxation^59^ or be a displacement behaviour^60^ and could, in this case, indicate stress recovery following disturbance. There could also be possible counteracting effects of providing both choice and change. Campbell et al.^54^ obtained inconsistent results in a spatial task when chicks were exposed to repeated introduction of novel objects, relocation of structures, and unpredictable sounds and lights in the rearing environment between 4 and 21 days of age^54^. Disturbance from the relocation of resource variants in the Changing*Multi-choice treatment, as with the disturbance from multiple stimuli in Campbell et al.^45^, may have interfered with the enhancement of spatial skills otherwise expected in a spatially complex environment. In future studies investigating effects of change and choice, an option might be to exchange rather than relocate the resource variants in the Multi-choice environment, making the type of change comparable with the change in the Single-choice environment. This alternative design, however, will require a greater number of variants of each resource and, additionally, different groups of chicks in the Single-choice environment will need to experience each of these variants to avoid confounding change and choice with differences in the variant types used in each treatment.

Previous studies investigating effects of environmental enrichments or more complex early environments, have included novelty (corresponding to our changing treatment) and spatial structures (corresponding to our choice treatment), either alone^15,16,61^ or at the same time^13,54^. Our findings suggest that these are two different types of inputs that affect chicks in different ways and, importantly, interact with each other. Further, previous studies compare the effects of these environmental enrichments to barren environments, whereas our treatments all provided basic resources such as litter and perches. This enabled us to focus specifically on the effects of environmental change and choice, thereby affecting predictability and controllability, respectively.

## Conclusions

The experience of environmental change, from exchanging litter and perch types, or of environmental choice, from presenting a variety of litter and perch types, could be advantageous for chick welfare during rearing because of an increase in active engagement with the environment. Further, a higher level of change reduced initial fear responses when first placed in a novel environment, while a higher level of choice stimulated exploration and spatial skills. However, an increase in both environmental change and choice, by relocating the resource types that provided choice, did not generally improve outcomes over those from change or choice alone. In fact, in some cases it resulted in intermediate outcomes or results no different from the standard condition offering neither change nor choice. These results suggest that change and choice have different but generally positive outcomes for the development of behavioural adaptability when provided in moderation. Based on these results, we encourage further studies on the benefits of providing change and choice during rearing for animals that will subsequently be transferred to a different environment.

## Methods

The study was approved by the Animal Research Ethics Committee in Uppsala SE (Protocol number 5.8.18-11549/2017). All methods were carried out in accordance with relevant regulations and following the ARRIVE guidelines^62^.

### Birds, housing and treatments

A total of 332 day-old female chicks of the white layer hybrid Bovans Robust from a commercial hatchery were allocated to 16 rearing pens (20 chicks/pen) in one room at the Swedish Livestock Research Centre (Lövsta). Average chick weight per pen was 33 ± 0.17 g. Four pens, balanced across room locations, were assigned to each of four treatments according to a 2 × 2 factorial design with two levels of environmental change (Non-changing/Changing) and two levels of environmental choice (Single-choice/Multi-choice). The treatments were, thus, Non-changing*Single-choice, Non-changing*Multi-choice, Changing*Single-choice and Changing*Multi-choice (Fig. 1). Seven extra chicks, temporarily kept in the standard treatment (Non-changing*Single-choice), replaced weak chicks during the first three days, after which the group composition remained the same until the study ended when the chicks were 5 weeks old. The remaining extra five chicks were reared in a spare pen and adopted out along with the experimental birds at the end of the study. Each pen was 1.2 × 2.4 m, with ad libitum feed (commercial starter type) and water located at one end. Perches were initially 25 cm from the ground and raised to 43 cm at 15 days of age. Lighting schedules and temperature were according to breeding company recommendations. Routine animal care occurred twice daily.

### Manipulating environmental choice and change

**Figure 5:**
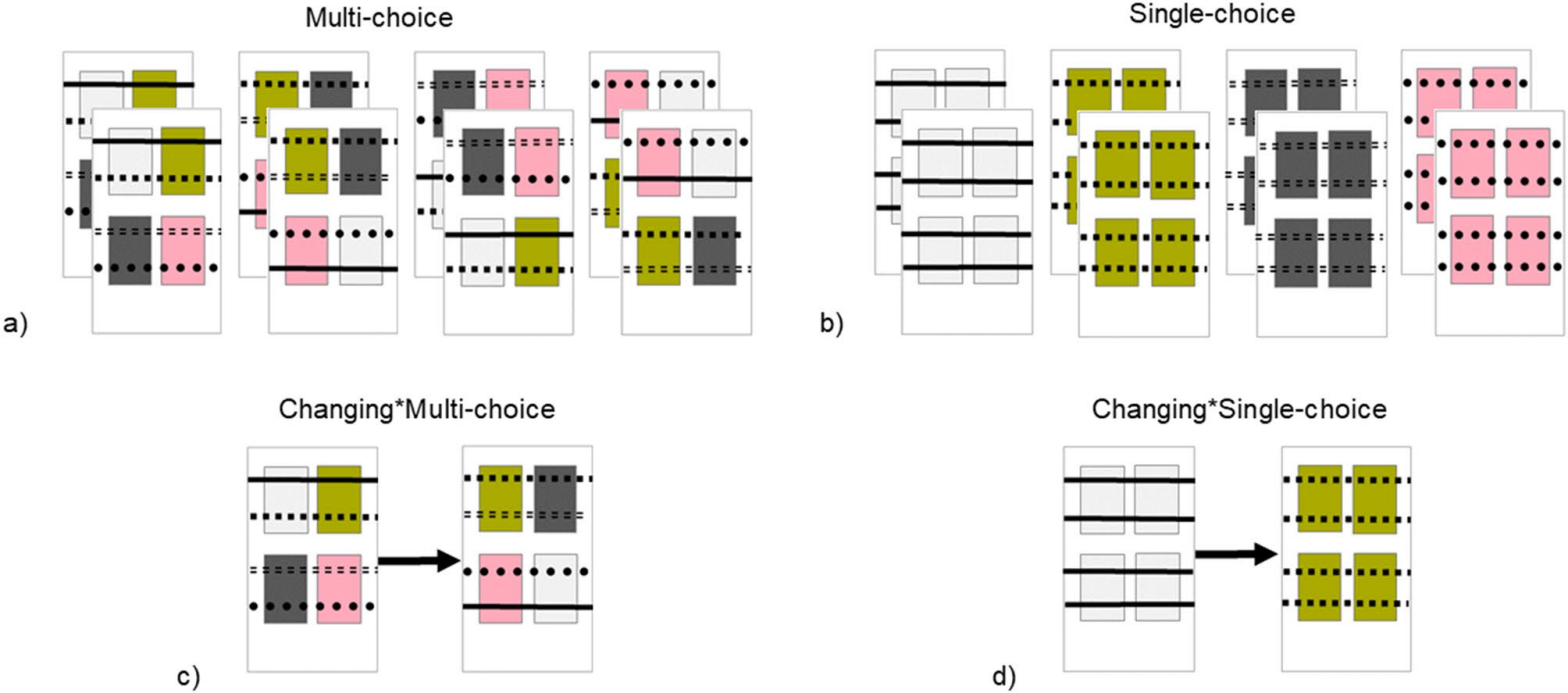
Each pen (N = 16) had four different locations for litter (coloured quadrants) and perches (shaped lines). Choice was created by varying the number of litter and perch types present in the pen, where (**a**) Multi-choice pens had different types of litter and perches placed in each of the four locations in the pen, while (**b**) the Single-choice pens had the same type of litter and perch in all four locations. The effect of specific litter and perch types was controlled by balancing the litter and perch types across locations in the Multi-choice pens and across pens in the Single-choice treatment. Change involved (**c**) rotation of the litter and perch type locations within Multi-choice pens or (**d**) exchange of one type of litter and perch to another type within Single-choice pens, according to a balanced design. Change occurred three times weekly in Changing pens.

Litter was presented in trays (70 × 35 × 3 cm) at four locations in each pen (Fig. 5) and kept in place by wooden frames equipped with 10 cm vertical plastic barriers to prevent litter from being mixed between trays. Four perches, each 120 cm in length, were placed at four locations in each pen (Fig. 5). Multi-choice pens (N = 8, Fig. 5a) had four different types of litter and four different types of perches, while Single-choice pens (N = 8, Fig. 5b) had a single type of litter and perch in all locations. The four litter types (straw, wood shavings, peat and sand) and perch types (rope, wood plank, rubber and wire) used in this study are described further in the Supplementary Information (Fig. 1 S1). Effects of specific litter and perch types were taken into account by balancing their use across treatment replicates since the aim was to compare Multi- and Single-choice treatments, not the effects of particular litter or perch types per se. A low stocking density (6.94 chicks/m^2^), along with ample litter and perch space per chick, made it possible for all chicks in the pen to use the same litter tray or perch at the same time if they chose to do so and also to better allow the expression of behavioural differences.

For the Non-changing environments, the set up with litter and perch types was the same throughout the study (Fig. 5a,b). For the Changing environments, litter and perch types were changed on eight occasions between 4 and 21 days of age, separated by one or two undisturbed days. Figure 5c illustrates an example of a change in a Multi-choice pen, involving moving locations of the litter and perch types. This was repeated on subsequent changes, until all litter and perch types had been in all possible locations. Figure 5d shows an example of a change in a Single-choice pen, involving replacing the litter and perch type with a different type. In subsequent changes, they were replaced again until all types had been presented. In both cases the process started again once all combinations were exhausted. Though this involved a different form of change in the Multi-choice and Single-choice pens (relocation of the existing types versus replacement with a new type), it allowed us to manipulate the same four variants of each resource. Further, while the birds may have experienced a change to or from a preferred variant or location as more or less positive, respectively, all birds experienced all variants. Had we exchanged the four litter and perch types in the Multi-choice pens with four new types of each, this could have introduced a possible bias as these new types would have only been experienced in this treatment. Experimenters entered the Non-changing pens at the time of change in the Changing pens to control for the effect of human disturbance.

### Behavioural observations during rearing

Live behavioural observations were conducted in the rearing pens between 4 and 22 days of age. Behaviours with a potential link to stress, comfort and environmental engagement were selected for the live observations (see Table 2 for overview and definitions). Each observation round comprised; (1) a scan for the number of chicks vigilant, the number moving, the number at each location (perch, litter tray or feeder) and the number engaged in preening, foraging, dustbathing, resting and feeding); (2) a one-minute continuous observation for pecking towards conspecifics (severe feather pecking and aggressive pecking) and; (3) a one-minute continuous observation of play behaviour (worm running, frolicking and sparring). Observations of feeding and resting were used to investigate behavioural synchronization and not analysed further for treatment differences. There were insufficient aggressive pecks for statistical analysis.

**Table 2:** Ethogram of behaviour observed in the rearing pens.

Behaviour	Definition	Average values per pen per observation
*Scan*		
Vigilant	Neck lifted or stretched with either fixed gaze or looking around, holding this posture for > 2 s. On perch or ground, sitting or standing, but not moving. Registered as proportion of chicks vigilant per scan. Excludes all other behaviours (apart from spatial clustering)	0.26 ± 0.12
Preening	Chick directs its beak to plumage on its own body (thorax, abdomen, shoulder, interior and exterior wings, rump, back, or cloaca) and carries out pecking, nibbling, combing or rotating movements once or rapidly. Definition from^63^. Registered as proportion of chicks preening per scan	0.11 ± 0.12
Foraging	Pecks directed to the litter while standing or walking or scratching the ground with the body bent forward while making a backward stroke with one leg. Definition from^64^. Registered as proportion of chicks foraging per scan. Does not exclude moving	0.09 ± 0.12
Dustbathing	While lying or squatting, chick performs dustbathing components (bill raking, vertical wing shakes, side lying, rubbing, scratching, ground pecking, and feather ruffling). Definition from^65^. Registered as proportion of chicks dustbathing per scan	0.02 ± 0.05
Moving	Locomotion, moving from one location to another propelled by leg and wing movements. Registered as proportion of chicks moving per scan. Can be combined with other behaviours, such as foraging and sparring	0.14 ± 0.12
Resting	Sitting or lying down with eyes closed or open and not dustbathing. Registered as proportion of chicks resting per scan	0.43 ± 0.32
Feeding	Pecks directed at food in the feeder. Registered as proportion of chicks feeding per scan	0.19 ± 0.19
Spatial clustering	The highest proportion of chicks at same location (at either same perch, same litter tray or at the feeder) per scan. Can be combined with all other behaviours	0.55 ± 0.19
Behavioural synchronization	The highest proportion of chicks performing the same behaviour (either preening, dustbathing, resting, foraging, feeding) per scan	0.62 ± 0.10
*One-minute continuous observation for pecking towards conspecifics*		
Severe feather pecks	Pecks at a conspecific that cause damage to the feather or a reaction by the receiver. Each peck was counted per 1 min observation	0.033 ± 0.20
Aggressive pecks	Pecking with force, almost always towards the back of the head/neck of the receiver. Each peck was counted per 1 min observation	0.009 ± 0.09
*One-minute continuous observation of play behaviour*		
Sparring	Play behaviour. Chick quickly approaches another chick with its breast somewhat elevated and bumps against or stops close to the other chicks’ chest. Each sparring chick was counted per 1 min observation. Same chick got more counts if there was a pause of 5 s between each sparring event	0.27 ± 0.97
Worm running	Play behaviour. Chick is running with something in its beak and may have running followers. Each worm running chick (excluding followers) was counted per 1 min observation. Same chick got more counts if there was a pause of 5 s between each worm running event	0.05 ± 0.33
Frolicking	Play behaviour. Chick makes a sudden twirl or run and may have running followers. Each chick displaying frolicking was counted per 1 min observation. Same chick got more counts if there was a pause of 5 s between each frolicking event	1.42 ± 3.98

The table presents average values for each observed behaviour per pen (mean ± SD).

On observation days, there were 4–8 rounds of behavioural observations. All pens were observed in each round, with at least 20 min between successive rounds of the same pen to reduce dependency between rounds. There were 2–4 rounds under undisturbed conditions (within a 4-h time period). Then the birds were disturbed by a person entering the pen, and either changing the litter and perches (Changing pens) or making no change (Non-changing pens). After this, there were 2–4 rounds under the relatively disturbed conditions (within the following 4-h time period). See Supplementary Information (Fig. 2 S1) for an overview. In total, 56 rounds of observations per pen (28 Undisturbed and 28 Disturbed) were conducted by two trained observers, with treatments and pens balanced between observers each day. Apart from training together on live behavioural observations, inter and intra reliability measures from video recordings were 14.47 and 14.87 CV%, respectively.

### Novel pen test

**Figure 6:**
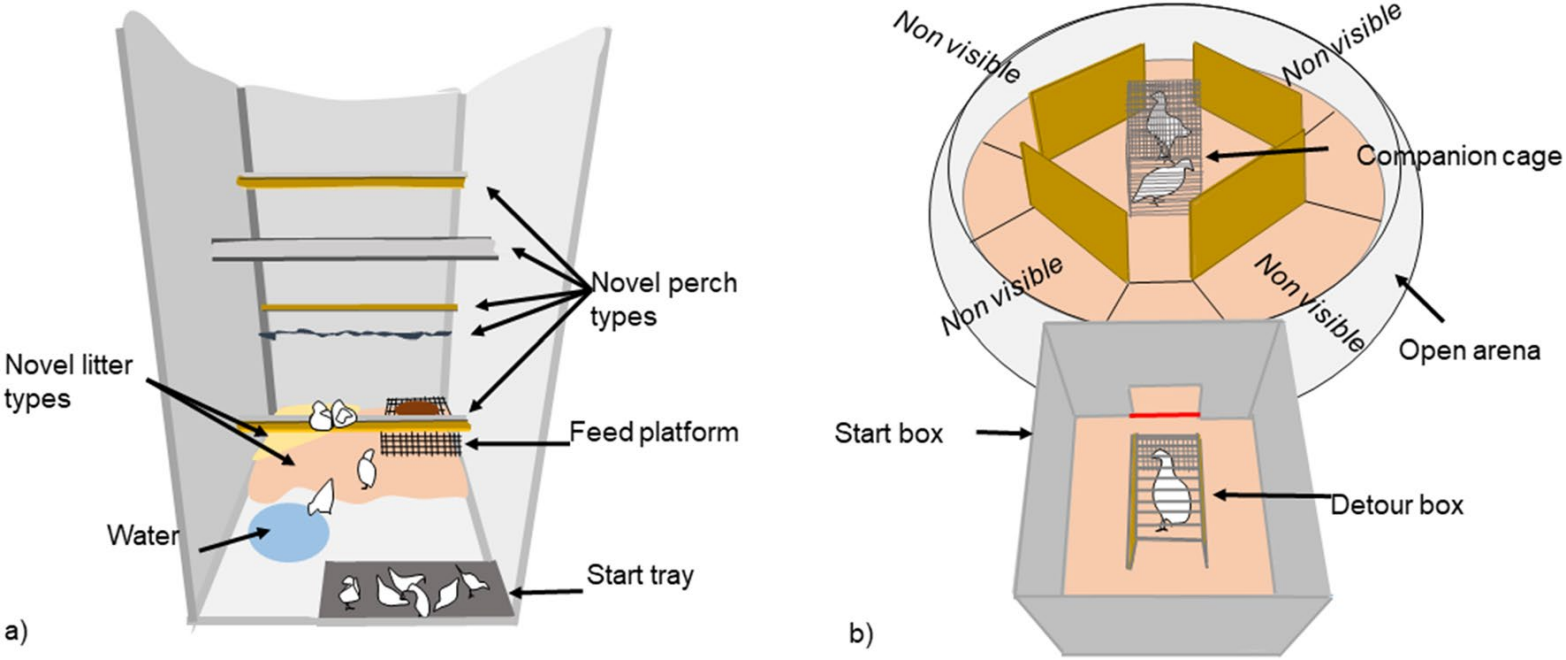
Chicks’ behavioural adaptability was investigated in two novelty challenges, (**a**) a Novel pen and (**b**) a Multivariate behavioural test. In the Novel pen, chicks were tested in familiar groups of ten following a transition to a novel pen (1.15 × 1.5 m) with novel resources or novel ways of presenting resources. These were a novel water container, a novel feed platform, two novel litter types (crushed straw pellets and ripped toilet paper) and four novel perch types (plastic mushroom shaped, thin rope, round wood, and a suspended piece of fabric). The pen had a concrete floor and walls covered with paper. In the Multivariate behavioural test, a chick had to first solve a detour task, as she could initially only see her pen mates through a wire net in the detour box (30 × 15 × 28 cm), and find her way out of the start box (55 × 55 × 50 cm) crossing the imaginary red line, to reunite with two pen mates (housed in the companion cage 30 × 50 × 35 cm) in the open arena (170 cm in diameter). The open arena had cardboard partitions that blocked the view of the companion cage. All flooring was covered with an unfamiliar litter type, hemp shavings. The whole test arena was covered with white net material.

This test was conducted when chicks were between 28–33 days of age. Chicks from each pen were randomly allocated to two groups of 10 chicks (from the same pen), and placed in two specially designed holding boxes. Four of these holding boxes (one per treatment combination) were then placed just inside the entrance of each of four novel pens (Fig. 6a). At the start of the test, the top and sides of the boxes were simultaneously lifted off, leaving the chicks standing on the floor of the box (dark-grey square in Fig. 6a). During the 1-h test, instantaneous scans were made of each group at 30 s intervals to determine (1) the number of chicks moving per pen (Table 2), and (2) the number of chicks located at each novel resource (litter, feeding platform and perches) (Fig. 6a), analysed as the average proportion of chicks at different novel resources per scan. Each observer alternated observations between two pens, balanced across treatments. At the end of the test, the chicks were returned to their rearing pen and the remaining 10 chicks per pen were tested. This testing procedure was repeated with the remaining three blocks of pens, giving a total of 16 h of data per treatment. The treatment groups were allocated to the four novel pens according to a balanced design.

### Multivariate behavioural test

The second novelty challenge test was conducted between 29–36 days of age, always at least one day after the Novel pen test, and according to procedures used previously for chicks of this age^13^. It involved testing chicks individually in a detour task and open arena (Fig. 6b). Six randomly selected chicks from each pen were placed in a transport crate. After 30 min of habituation in the test room, three chicks were placed in the companion cage located in the middle of the open arena (Fig. 6b). After a 3-min habituation period, one of these chicks was taken out and placed in the detour box where she could see her pen mates through a wire net. To reach her two companions, the chick needed to walk in the opposite direction to exit the detour box and then find her way out of the start box (crossing the imaginary red line in Fig. 6b). If a chick did not leave the detour box within 7 min, the box was removed in order to make the task easier. If not leaving the start box within 10 min, the chick was gently pushed out through the exit. After leaving the start box, 5 min was given to explore the open arena, after which the chick was exchanged with a chick in the companion cage until all three chicks had been tested. The test was then repeated with the other group of three chicks from the same pen. Two experimenters, each with their own tasks, worked together to conduct the test. Behavioural observations involved recording of (a) duration of initial freezing after being placed in the detour box (latency to show head movements, standing up or moving legs) reflecting an initial fear response; (b) latency to solve the detour task and leave the start box (counted from the end of the first freezing bout) reflecting spatial skills, and; (c) number of lines (12 black lines) crossed in the open arena (see lines in the illustration) during the 5-min period, reflecting movement, as an indication of exploration. Crossing lines also meant moving into areas where the birds in the companion cage were not visible.

### Statistical analysis

Statistical analyses were processed in RStudio (Version 1.3.959). Counts and latencies were modelled using a linear mixed model fit by REML and the Satterthwaite’s method (lmertest). Proportion data were analysed using generalized linear mixed models with a binomial distribution using the glmer-function of the lme4-package. All models included Change, Choice and the interaction between Change and Choice as fixed effects and Rearing Pen ID as a random effect. The effects of observer and observation order were not significant for any variables, and were therefore excluded from all models. Models for data from the rearing observations included the fixed effects of age (week), Disturbance and a three- (and two-) way interaction between Change, Choice, and Disturbance. Full models for data from the Multivariate behavioural test included chick and group test order. These were kept if having a significant effect (chick for freezing duration), or otherwise removed to simplify models (latency to solve detour, number of lines crossed). Assumptions were visually checked by residual plots; normal probability plot of residuals (QQ plot) and residuals versus fitted values. If models failed to converge, the random effect of pen was removed and weekly pen means were analysed with week as a fixed effect to control for repeated measures (behaviour in the rearing pens: vigilant, preening, foraging, dustbathing, moving, resting, spatial clustering, behavioural synchronization) or group means with group as a fixed effect (Novel pen test: moving, proportion of birds at novel resources). Overall pen means were analysed if necessary to achieve model convergence (severe feather pecking, sparring, frolicking). If model assumptions were not met, a Kruskal Wallis test was performed on overall pen means (worm running). Effects were explored using a Type III Analysis of Variance Table with Kenward-Roger's method for linear mixed models, while the Anova-function in the car-package was used for linear models or generalized models. Interactions of Change and Choice were investigated using estimated marginal means from the emmeans-function. A significance level of 0.05 was used and trends were ignored. In the presence of significant pairwise-comparisons of interactions, significant main effects were ignored. All results from our fitted models are presented as estimated marginal means and standard error of the estimated mean, whereas data investigated using the Kruskal Wallis test are presented by means (Aggregate function) and standard error of the mean. Log-transformed values (freezing duration, latency to solve detour task) are presented as back-transformed to the original scale.

## Supplementary Information

Supplementary Information.

## Data Availability

The datasets generated during and/or analysed during the current study are available from the corresponding author on request.
