# Supplementary material for: Environmental change or choice during early rearing improves behavioural adaptability in laying hen chicks
Source: Sci Rep. 2023 Apr 15;13:6178. doi: 10.1038/s41598-023-33212-0 (PMC10105694; doi:10.1038/s41598-023-33212-0)
Supplement: Supplementary file 1 — Supplementary Information. [file 41598_2023_33212_MOESM1_ESM.docx]

Table 1. Behaviours with no significant treatment differences (mean±SE), where the observations in the rearing pens are divided between observations before (Undisturbed) and after (Disturbed – in italics and shaded with purple) the environmental change or human visit alone. The significant comparisons are shown in the manuscript.

|  | 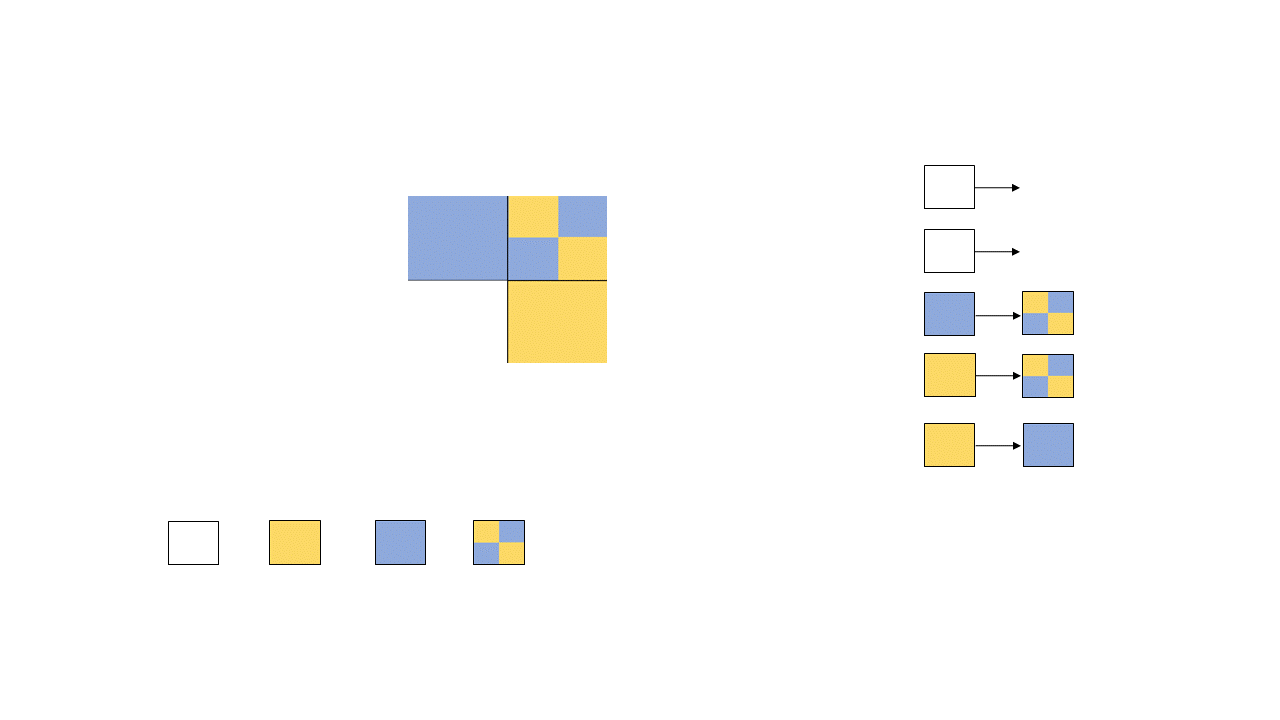Non-changing  *Single-choice | 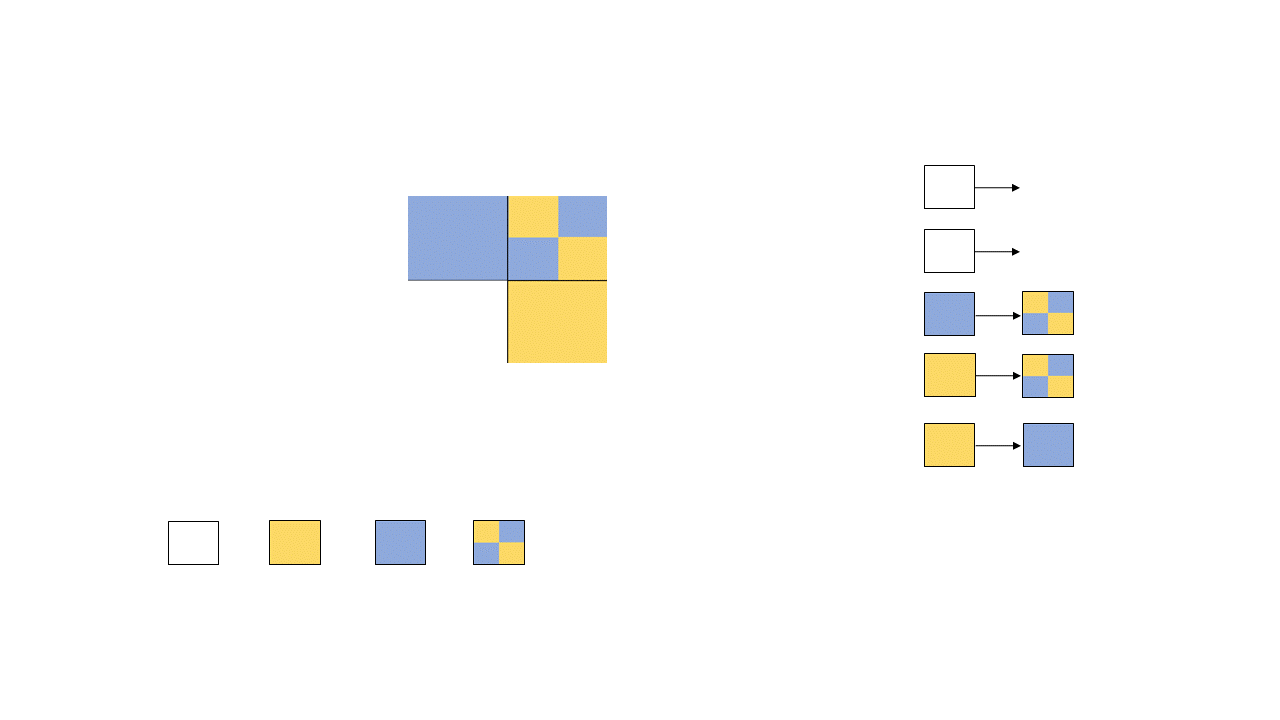Changing  *Single-choice | 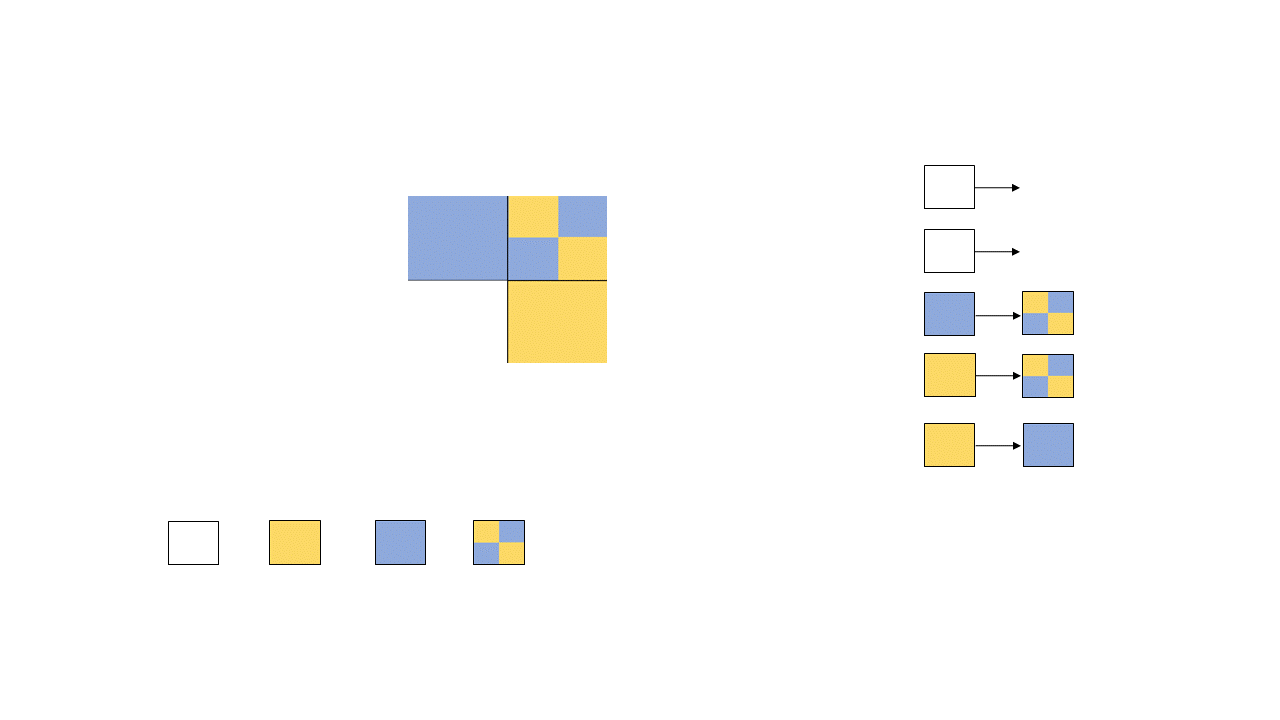Non-changing  *Multi-choice | 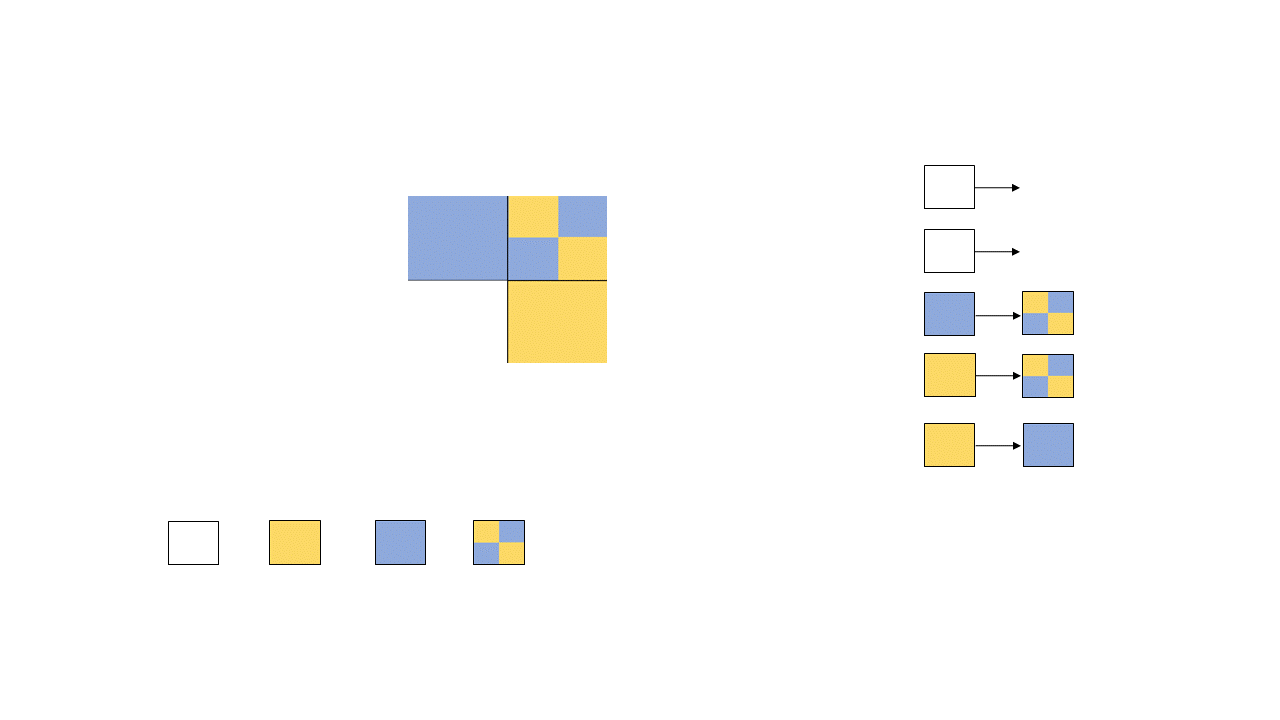Changing  *Multi-choice |
| --- | --- | --- | --- | --- |
| *Scan sampling (proportion of chicks performing behaviour/scan)* | | | | |
| Vigilant | 0.29±0.03 | 0.28±0.03 | 0.29±0.03 | 0.27±0.03 |
| *Vigilant* | *0.26±0.03* | *0.26±0.03* | *0.20±0.02* | *0.21±0.02* |
| Preening | 0.10±0.01 | 0.10±0.01 | 0.10±0.01 | 0.10±0.01 |
| Foraging | 0.09±0.01 | 0.07±0.01 | 0.10±0.01 | 0.10±0.01 |
| Dustbathing | 0.02±0.00 | 0.02±0.00 | 0.01±0.00 | 0.02±0.00 |
| *Dustbathing* | *0.03±0.01* | *0.03±0.01* | *0.01±0.00* | *0.02±0.01* |
| Moving | 0.17±0.03 | 0.13±0.02 | 0.12±0.02 | 0.11±0.02 |
| Behavioural synchronization | 0.60±0.02 | 0.60±0.02 | 0.60±0.02 | 0.63±0.02 |
| *One-minute continuous observation for pecking towards conspecifics (counts/min/20 chicks)* | | | | |
| Severe feather pecking | 0.04±0.02 | 0.02±0.02 | 0.05±0.02 | 0.02±0.02 |
| *Severe feather pecking* | *0.03±0.02* | *0.02±0.02* | *0.06±0.02* | *0.03±0.02* |
| *One-minute continuous observation of play behaviour (counts/min/20 chicks)* | | | | |
| *Sparring* | *0.22±0.08* | *0.14±0.08* | *0.29±0.08* | *0.30±0.08* |
| Worm running | 0.03±0.02 | 0.06±0.02 | 0.14±0.12 | 0.13±0.06 |
| *Worm running* | *0.19±0.1* | *0.30±0.13* | *0.27±0.23* | *0.13±0.07* |
| Frolicking | 1.37±0.35 | 1.62±0.35 | 1.77±0.35 | 2.14±0.35 |
| *Frolicking* | *1.63±0.35* | *1.09±0.35* | *0.91±0.35* | *1.29±0.35* |
| *Novel pen challenge test* | | | | |
| Proportion of chicks at novel resources | 0.16±0.06 | 0.19±0.06 | 0.18±0.06 | 0.19±0.06 |

### Age effects

Age was included in the statistical models for behaviour in the rearing pens. As expected, there were significant effects for many of the behaviours. With increasing age, there was less spatial clustering (z=-4.27, P<0.001) and behavioural synchronization (z=-3.45, P<0.001), a lower proportion of chicks foraging per scan (z=-3.13, P=0.002) and a higher proportion of chicks preening (z=3.79, P<0.001) and moving (z=1.97, P=0.049) per scan. These general age effects are not discussed further.


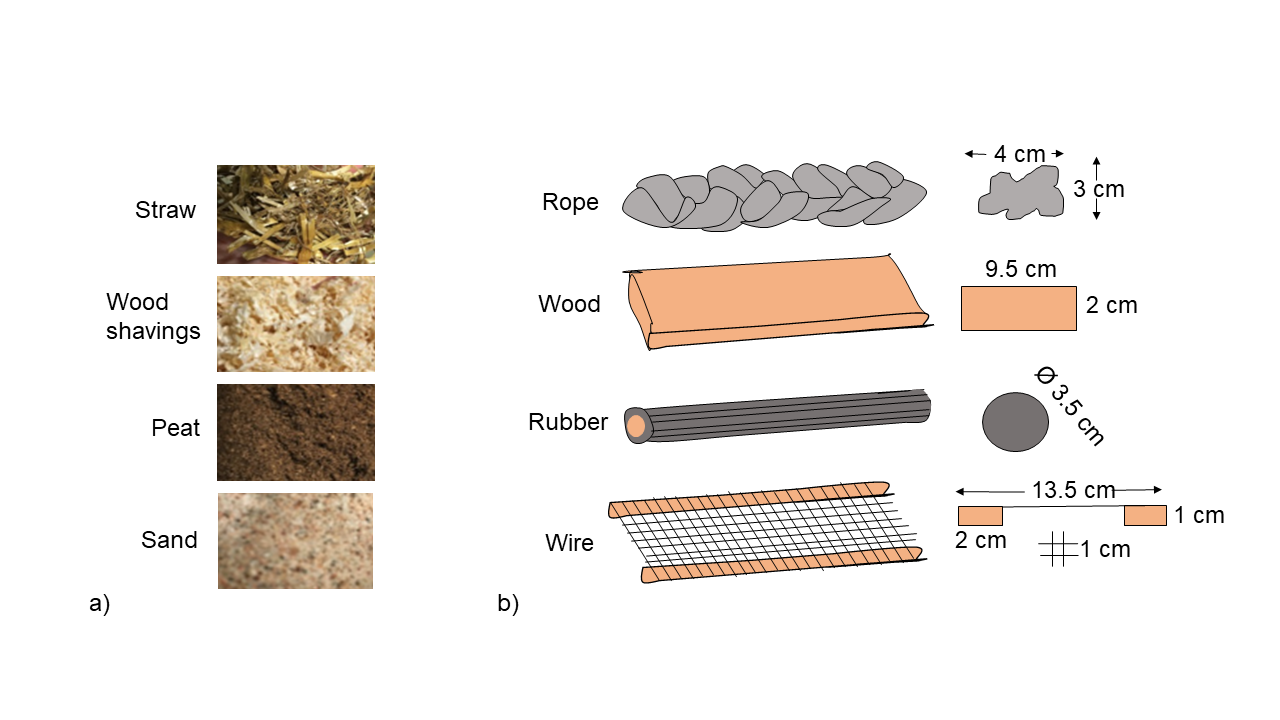


Figure 1. The four different litter and perch types used in the study. The four different litter types were included based on apparent differences in structure, particle size, sensation and smell. On the left (a) pictures show the four different litter types used: straw, wood shavings, peat and sand. The four perch types were chosen based on differences in structure, shape and sensation but also on the different bodily actions needed to land and move on them, ensuring an increase in structural complexity. Illustrations to the right (b) show the side and end views, including dimensions of the different perch types: braided rope, flat wood plank, round rubber (ribbed rubber around a wooden pole core) and flat wire (with a wooden frame).


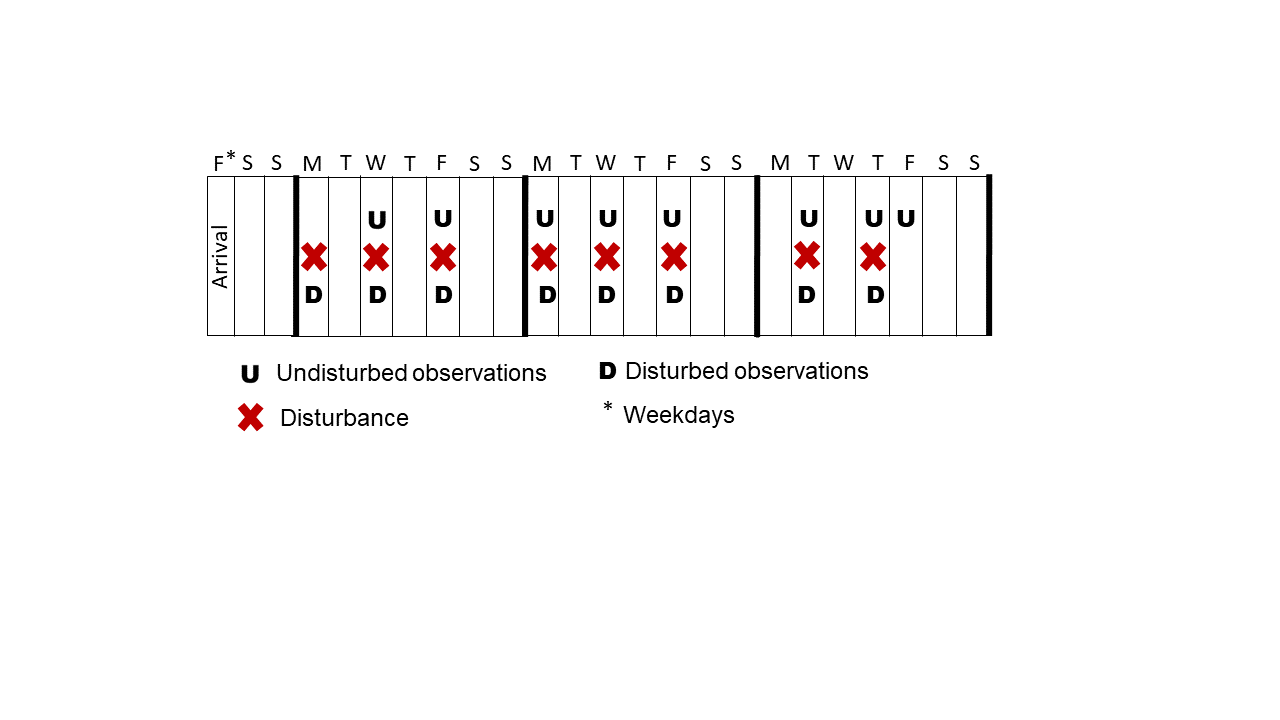


Figure 2. A schematic showing disturbances which occurred in the rearing pens on 8 occasions during the first four weeks after hatch, whereby a person walked inside the pen, with (in changing pens) or without (in non-changing pens) changing litter and perches. Behavioural observations were made during 2-4 rounds before (undisturbed - U) and/or after (disturbed - D) each disturbance.
